# Exploring sustainability in dairy cattle breeding focusing on feed efficiency and methane emissions*

**DOI:** 10.3168/jdsc.2023-0461

**Published:** 2024-03-29

**Authors:** C.M. Richardson, J.J. Crowley, B. Gredler-Grandl, P.R. Amer

**Affiliations:** 1 AbacusBio International Ltd., Edinburgh, UK, EH25 9RG; 2 AbacusBio Ltd., Dunedin, New Zealand, 9016; 3 Wageningen University & Research Animal Breeding and Genomics, 6700 AH Wageningen, the Netherlands

## Abstract

Reducing emissions is vital to improve sustainability, and industry leaders have set emission goals to reduce gross emissions, lower emissions intensity, or reach net zero. However, additional traits should also be measured and compared in terms of their impact on the broader definition of sustainability. In addition to environmental impact, a sustainable breeding objective must consider profit, animal welfare, farmer well-being, and social responsibility. Traits to be considered include direct emissions (e.g., nitrogen and methane), production efficiency (e.g., feed efficiency), health (e.g., calf and transition cow health), and welfare traits (e.g., polled). Many of these novel traits require labor- and cost-intensive phenotyping procedures. Consequently, this results in relatively modest data sets and estimated breeding values with limited reliability. Opportunities exist to overcome this limitation by developing cost-effective and easily quantifiable proxy traits and utilizing international collaboration to expand novel phenotype reference populations. Furthermore, noneconomic values can be estimated to quantify the impact of a trait on societal perspective (e.g., farmer preference) or environmental impact (methane emissions), and combined with economic weights to calculate aggregate weights for each trait. Although validation techniques are still uncertain, the United Nations Sustainable Development Goals may be applied to determine the improvement in sustainability due to genetic selection. This approach provides the flexibility to accommodate diverse sustainability perspectives, encompassing the disparities between developed and developing countries. Currently, the number and quality of relevant phenotypes are the main limiting factor. However, as confidence grows in the opportunity to improve sustainability through genetic selection, substantial new investment will be required in both refining phenotyping methodologies and conceptualizing novel breeding designs.

## Highlights

• A sustainable breeding objective must consider profit, animal welfare, farmer well-being, and social responsibility.
• Noneconomic values can be estimated to quantify the impact of a trait on societal perspective (e.g., farmer preference) or environmental impact (methane emissions), and can be combined with economic weights to calculate aggregate weights for each trait.
• The United Nations Sustainable Development Goals may be used to gauge improvements in sustainability due to genetic selection.

## Graphical Abstract

**Figure:**
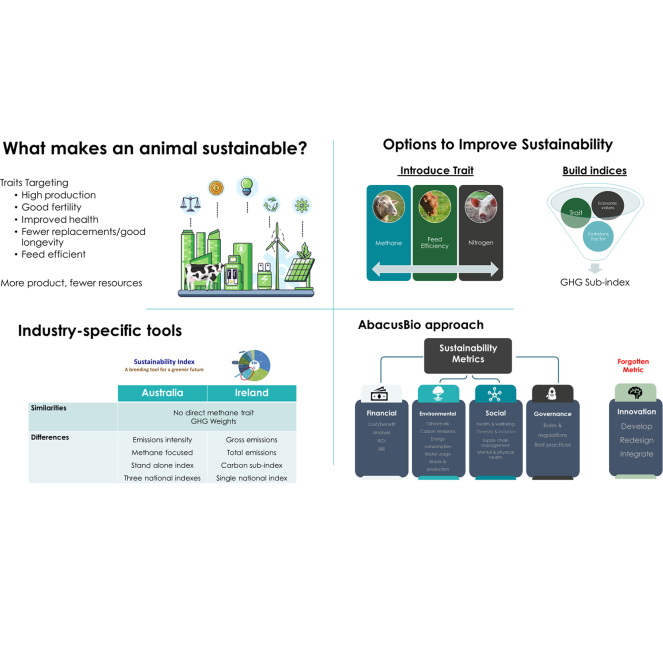
**Summary:** Sustainable animal production can be achieved by optimizing breeding programs to target traits that generate productive cattle that require fewer resources.

Current perspectives on sustainability largely focus on reducing greenhouse gas emissions to maintain global warming below 1.5°C and limit the detrimental effects of climate change (IPCC, 2018). In accordance with the updated 2016 Paris Agreement, governments have set national targets to reduce emission by 43% by 2030 and reach net zero by 2050 (UNCC, 2016). To meet these government standards, dairy industries have pledged to reduce gross emissions (ICBF, 2020), reduce emissions intensity (Dairy Australia, 2021), or reach net zero emissions (Dairy Farmers of Canada, 2021). Due to these aggressive targets and social perspectives, immediate focus needs to be given to reduce emissions, while maintaining progress in other biological traits impacting long-term sustainability. A robust definition of sustainability involves every aspect of the commodity chain and affects the entire production system, from the dairy cow and the farmer to the consumer and the larger societal perception.

The International Committee of Animal Recording (**ICAR**) has established a Sustainability Task Force to propose a list of traits for the purpose of harmonizing the approach to assess the sustainability of dairy herds (ICAR Sustainability Task Force: Composition and produced documents; Roalkvam et al., 2023). Traits were selected to cover the most important aspects of herd performance regarding sustainability. The list contains several categories of traits, including (1) feeding and production, (2) fertility, (3) health, (4) longevity and culling, and (5) young stock. However, the method to develop a sustainability index or alternative management tool is still under investigation with international organization developing partnerships to solve such issues. As a component of the sustainability task, the ICAR Feed and Gas Working Group supported Dr. Caeli Richardson as the inaugural participant of the Brian Wickham Young Person Exchange (**BWYPEX**) Program (https://www.icar.org/index.php/about-us-icar-facts/brian-wickham-young-person-exchange-program-bwypex-consideration-by-potential-partners/).

The BWYPEX Program was initiated to create opportunities for young scientists to interact with host organizations internationally. Of proposed topics, improving sustainability of cattle was promoted by the ICAR Feed and Gas Working Group as a strong area of global interest. Feed efficiency and traits related to environmental impact, such as methane emission, are key traits in animal production to increase sustainability on animal, herd, and system levels. The program aimed to explore the current implementation of sustainability traits, as well as possibilities for routine recording and monitoring services.

Topics investigated within the scope of the BWYPEX Program included identification and definitions of efficiency and environmental traits, recording techniques, available proxies, and possible tools in recording services to increase sustainability. Information was collected using unstructured interviews and focus groups. This methodology was used to support the exploratory nature of the program and enable the collection of a combination of qualitative and quantitative data to gain an understanding of current perspectives on breeding for sustainability, as well as to reveal future research ideas (Thelwall and Nevill, 2021). Initial invitations for interviews were extended to organizations participating in the BWYPEX program and active members of the ICAR Feed and Gas Working Group. Interviews were conducted in person and virtually over a 6-mo period. In-person host organizations included Wageningen University & Research (the Netherlands), Lactanet (Canada), AbacusBio Ltd. (New Zealand), the National Institute for Agricultural and Food Research and Technology (INIA, Spain), Aarhus University (Denmark), and the Irish Cattle Breeding Federation (ICBF, Ireland). Additional interviews were completed virtually with Agriculture Victoria (Australia) and Walloon Agriculture Research Centre (Belgium). During the collaboration, industry partners, academic institutions, and commercial farms were visited to gain a multilevel understanding of factors that impact sustainability and potential widespread mitigation strategies.

Through the international collaboration, an overview and evaluation of traits and proxies relevant for sustainable dairy production was completed. Overall, key opportunity areas highlighted for improving sustainability in dairy cattle and innovating the industry included increasing data availability, defining robust breeding objectives, and expanding interdisciplinary research.

Data availability continues to be a major limitation in breeding animals for sustainability; however, the scope of this challenge has changed over time, particularly for feed efficiency and methane emissions (Basarab et al., 2013; Difford et al., 2020; van Staaveren et al., 2023). For example, initial methods for recording feed intake and methane emissions were expensive and labor intensive, and therefore it was nearly impossible to obtain records on commercial animals. Current research projects have developed technologies to overcome this, through updated equipment or data analysis methods (Vanlierde et al., 2021; Lassen et al., 2023).

Methane emissions may be predicted using mid-infrared (**MIR**) spectroscopy of milk samples collected during routine herd improvement testing, offering an inexpensive and relatively simple alternative to gold standard testing (Vanlierde et al., 2015). As MIR is routinely collected at herd recording, the data are freely available; however, to predict accurate phenotypes on commercial animals, a representative gold standard training population with sufficient variation is required (Vanlierde et al., 2021). Additionally, the development of sniffer technology, which can be directly installed in commercial milking robots, allows for accurate measurement of methane emissions on individual cows at each milking (Benzoni et al., 2023). This has led to the development of many methane phenotypes from commercial farms being available around the world.

Feed intake is more challenging; however, in Denmark, recycled equipment from the gaming industry has been adapted to provide individual cow estimates of feed intake (Lassen et al., 2023). A 3-dimensional camera technology to measure feed intake and BW on individual cows in commercial farms has been developed. The camera-based method for feed intake measurements has been validated using scale measurements and termed the Cow Feed Intake (**CFIT**) system (Manzanilla-Pech et al., 2023). Several management tools are being developed based on data from farms that have the equipment installed, and more traits are expected to be developed based on the 3-dimensional camera technology in the future.

Although technologies do exist for collecting methane and feed intake data on commercial herds, most inexpensive or less labor-intensive systems are limited to nonpastoral systems. This highlights the challenges of breeding for lower emissions and more efficient cattle in pastoral systems. Currently, in pastoral systems, feed intake records must be collected in research facilities either using a proxy diet, such as the hay cube fed in Australia, or by manually cutting and individually filling feed bins. Keeping in mind that the average dairy animal consumes approximately 20 kg of DMI per day (Manzanilla-Pech et al., 2021), this is an extremely labor-intensive method and unsustainable on commercial farms. Using CFIT cameras in pasture is currently not an option due to the interaction of sunlight with the cameras' sensors creating a blind spot in camera data. A similar circumstance exists when considering inexpensive methane phenotypes, as rarely do pastoral systems use milking robots with sniffers installed. Several studies have explored methods of estimating feed intake in pasture-based systems, with the n-alkane technique emerging as a promising proxy, particularly for individual dairy cows. This technique demonstrated consistent accuracy in estimating herbage intake across varying herbage masses and seasons (Wright et al., 2019). Furthermore, advancements in sensor technology, such as the RumiWatch System, have shown promise in accurately identifying prehension bites in both grazing and stall-fed cows (Norbu et al., 2021).

One of the continuing challenges in defining a robust breeding objective is valuing traits with low direct economic value. This includes traits such as animal welfare or emissions, where the true value of their improvement is not reflected only through economic gain. Although these traits are generally valued through a desired gains approach by calculating the index emphasis required to achieve the desired genetic gain, additional methods should be considered that directly relate to sustainability. For traits without a direct economic value, noneconomic weights, such as those derived through the 1000minds survey approach devised by Martin-Collado et al. (2015), or a desired gains approach, may be used to apply additional emphasis on trait based on farmer preference or social impact. Another example of noneconomic weights are emissions coefficients, such as those described by Amer et al. (2018), which describe the change in emission per unit change in a trait. These different approaches to prioritizing traits have been combined to develop sustainability indexes for genetic selection purposes, such as the Australian Sustainability Index and the Irish Economic Breeding Index (**EBI**).

Each of these dairy industries have produced breeding programs that focus on the broader definition of sustainability, but the approaches taken to reduce emissions differ due to the specific goals and restraints of each dairy system (Table 1). Although the Sustainability Index and the EBI do not currently include direct methane breeding values in their national selection indexes and instead apply greenhouse gas coefficients to penalize traits based on environmental impact (Amer et al., 2018), major differences do exist. In Australia, the dairy industry has pledged to reduce emissions intensity. Contrastingly, Ireland has set a national target to reduce gross emission, which has also been applied to agriculture. Australia's index is centered on reducing methane emissions, whereas Ireland uses a total carbon emissions approach. As Australia has multiple national selection indexes, each designed to appeal to a unique subset of farmer needs, the Sustainability Index (DataGene Ltd., 2022) was introduced as a third national selection index. However, in Ireland, the emissions indexes have been directly inputted into the single national selection index through a carbon subindex. A comparison of these indexes highlights the importance of avoiding a single approach to defining sustainability-focused indexes and the necessity for system-specific strategic sustainability plans.

**Table 1:** Similarities and differences between sustainability-focused national selection indexes that include noneconomic weighting on traits using environmental coefficients

Component	Sustainability Index, Australia	Economic Balanced Index, Ireland
Methane trait	Does not include a direct methane trait	
Index weights	Applies a greenhouse gas weight to penalize traits based on environmental impact	
Breeding objective	Emissions intensity	Gross emissions
Emissions targeted	Methane emissions	Total carbon emissions
Implementation method	Independent index	Carbon subindex
National index strategy	Three national indexes	Single national index

The UN defines sustainability as development that meets the needs of present society without compromising the ability of future generations to meet their own needs (Brundtland and Khalid, 1987). In 2015, the United Nations presented 17 Sustainable Development Goals (**UN SDG**) with the plan to build an international partnership for sustainability (United Nations, 2022). These goals include aspects such as no poverty, zero hunger, good health and well-being, and quality education. Cumulatively, they recognize that actions in one area will affect outcomes in another, and that development must balance social, environmental, and economic sustainability.

Similarly, the definition of sustainability in general business development also follows an all-encompassing approach. In business, environmental social governance (**ESG**) plans are commonly used to determine the health of a company and to predict future success (Whitelock, 2019). These plans benchmark the outcomes of a company against key sustainability metrics broken down into 4 categories: financial, social, environmental, and governance (Figure 1).

**Figure 1:**
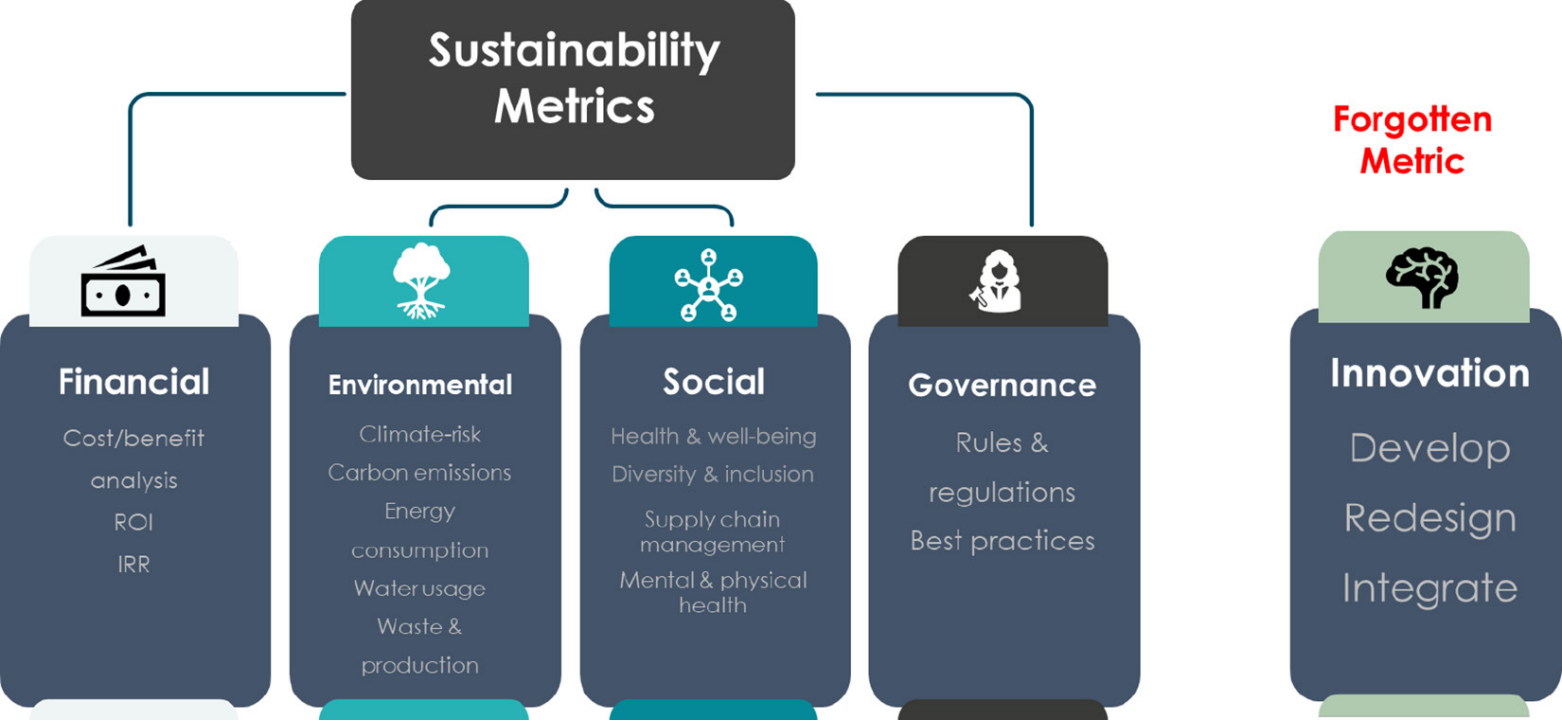
Sustainability metrics commonly used to develop business environmental social governance plans. These metrics aim to value the long-term success of a business beyond financial metrics such as return on investment (ROI) and internal return rate (IRR). Applying value to traits using these sustainability metrics, as well as innovation, may offer novel ways of developing selection indexes.

Although historically dairy farmers' management decisions may not have been driven specifically by these sustainability metrics, these pillars are cohesive with farm success. In dairy cattle, finances can be improved by increasing revenue (e.g., selecting for increased production), or through reducing costs (e.g., selecting for animals with higher genetic merit for health and survival traits). Environmental impact may be improved through reducing resource use (e.g., selection for lower emissions or higher feed efficiency). Taking into account the welfare of animals and farmers, strategic choices such as favoring traits like polled cattle or reducing inbreeding levels emerge as pivotal for enhancing social metrics. Governance parameters consider the industry's best management practices, regulations, or agreements in place with the breeding community to better manage inbreeding or welfare traits.

At its foundation, a sustainable animal is one that is high producing and requires minimal resources or farmer intervention. Although we can select animals that produce less emissions, it is vital that breeding objectives continue to drive the selection for high-producing, fertile animals that are healthy, have strong longevity, and use feed efficiently—essentially, animals that are capable of creating more products with fewer resources. For novel traits, much focus has been placed on defining and implementing methane emission traits. However, effective genetic mitigation strategies are dependent on the industry's specific emissions target (reducing gross emissions, lowering emissions intensity, or reaching net-zero emissions) and may include only a subset of opportunities to reduce emissions based on available mitigation tools.

Currently, inventory calculators fail to account for reductions that change the static emissions coefficients (i.e., methane per kilogram of dry matter) used to estimate per-animal individual emission output. Thus, achieving strict gross emissions reduction protocols may be possible only through large-scale culling and reducing of the national herds. Alternatively, an emission intensity reduction strategy proves most effective in reducing a national industry inventory if national production levels can be constrained at a fixed ceiling. In practice, at a policy level, it is very hard to constrain the national level of output and, given the rapidly increasing human population, in most countries it will be more sustainable to incentivize a shift toward the most efficient producers of animal products. Net Zero is an emissions target with the goal for an industry to mitigate the same level of emissions that it emits. This allows an industry to continue increasing production, as long as additional strategies are implemented to offset the corresponding increases in emissions.

Rarely is the impact of animal breeding and genetic selection measured beyond rates of genetic gain for economically important traits. However, closer consideration of the UN SDG demonstrates not only the wide impact of animal breeding but also the importance of considering more than emissions when defining sustainability (Table 2). By increasing farmer profitability and self-sufficiency, the industry works toward lowering poverty. With 27% of the global workforce being employed in agriculture as of the year 2021, production growth not only represents a push toward zero hunger but also increases the role of agriculture in modern economies to ensure decent work and economic growth. Animal breeding applies state-of-the-art genetic technology and data recording systems to positively impact industry, innovation, and infrastructure. Improving genetic gain supports responsible consumption and production by generating nutritionally dense food while creating high-quality long-lasting materials.

**Table 2:** The role of animal breeding on improving the United Nations Sustainable Development Goals (UN SDG)

UN SDG	Impact of animal breeding
No poverty	Increasing farmer profitability and self-sufficiency
Gender equality	Improving the role of female farmers in developing countries
Decent work and economic growth	Increasing the role of agriculture in economies
Industry, innovation, and infrastructure	Developing state-of-the-art genetic technology and data recording systems
Sustainable cities	Supporting self-sufficiency in developed and developing countries
Responsible consumption and production	Creating high-quality, long-lasting material
Climate action	Reducing emissions
Life below water	Decreasing water usage to rear and maintain animals, and managing wastewater
Life on land	Improving animal welfare and the well-being of people

Most impressively, animal breeding plays a vital role in boosting self-sufficiency and supporting gender equality in developing economies. Project Mesha, a collaboration with the Nimbkar Agricultural Research Institute (NARI) and the Aga Khan Foundation, focuses on building a genetic improvement strategy in India's Bhair region that allows the selection of breeding buck goats based on objective recorded criteria. This program improves the quality of life for marginalized landless people and empowers and raises the incomes of women who keep goats by improving the productivity of their goats using genetic selection and low-cost data recording. Improving production of goats in these areas supports social equality by increasing the role of women in developing economies. This is an attribute of sustainability that current validation processes do not consider.

Using a combination of UN SDG and ESG sustainability metrics allows each system to be considered individually. Additional challenges exist when determining the optimal method to combine these coefficients. Similar challenges are currently seen with the increasing amount of omics data in estimating animal genetic merit. Validation methods could be applied to determine the best way to apply a UN SDG coefficient weighting approach. More importantly, applying this ESG measurement technique would allow for attributes of the UN SDG to be considered in trait selection.

Introducing such a technique to measure the impact of selection on sustainability brings to questions the weight that each component of sustainability should receive and requires interdisciplinary collaboration

*Doughnut Economics*, by Kate Raworth (Raworth, 2017), introduces the idea of measuring a country's health based on its position within 2 boundaries: a social foundation and an ecological ceiling. This concept offers an alternative to productivity measures such as gross domestic product, by focusing on balancing the use of natural resources with basic human needs, to prevent overconsumption or social injustice. Applying a similar approach to animal breeding could be one option to combine sustainability weights beyond using economic values.

The concepts presented in this paper aim to provide a basis for developing a broader definition of sustainability, beyond the focus of reducing emissions. The development of new genetic tools and strategies to reduce emissions, such as novel traits and indexes, will require sufficient investment as well as international collaboration. Future research focused on defining region-specific optimal breeding objectives and comparing the impact of different selection strategies on broader sustainability metrics is required.
